# Amelioration of Radiation-Induced Cell Death in Neuro2a Cells by Neutralizing Oxidative Stress and Reducing Mitochondrial Dysfunction Using N-Acetyl-L-Tryptophan

**DOI:** 10.1155/2022/9124365

**Published:** 2022-11-26

**Authors:** Ravi Kumar, Pratibha Kumari, Swapnil Pandey, Shravan Kumar Singh, Raj Kumar

**Affiliations:** ^1^Division of CBRN, Institute of Nuclear Medicine and Allied Sciences, Defence Research and Development Organization, Brig. S.K. Mazumdar Road, Delhi 110054, India; ^2^Microbial Technology Department, CSIR-National Botanical Research Institute, Rana Pratap Marg, Lucknow, Uttar Pradesh 226001, India

## Abstract

The deleterious effects of ionizing radiation on the central nervous system (CNS) are poorly understood. Radiation exposure during an accidental nuclear explosion, nuclear war, or radiotherapy causes severe brain damage. As a result, the current work is carried out to assess the radioprotective potential of N-acetyl-L-tryptophan (L-NAT) in neuronal cells. Radiation-induced cell death and its amelioration by L-NAT pretreatment were investigated using MTT, SRB, CFU, and comet assays. Flow cytometric and microscopic fluorescence assays were used to investigate radiation-induced oxidative stress, alteration in mitochondrial redox, Ca^2+^ homeostasis, depolarization of mitochondrial membrane potential, and its prevention with L-NAT pretreatment. Western blot analysis of Caspase-3, *γ*-H2aX, p53, ERK-1/2, and p-ERK-1/2 expression was carried out to identify the effects of L-NAT pretreatment on radiation-induced apoptosis and its regulatory proteins expression. The study demonstrated (MTT, SRB, and CFU assay) significant (~80%; p <0.001%) radioprotection in irradiated (LD_50_ IR dose) Neuro2a cells that were pretreated with L-NAT. In comparison to irradiated cells, L-NAT pretreatment resulted in significant (p <0.001%) DNA protection. A subsequent study revealed that L-NAT pretreatment of irradiated Neuro2a cells establishes oxidative stress by increasing antioxidant enzymes and mitochondrial redox homeostasis by inhibiting Ca^2+^ migration from the cytoplasm to the mitochondrial matrix and thus protects the mitochondrial membrane hyperpolarization. Caspase-3 and *γ*-H2aX protein expression decreased, while p-ERK1/2 and p53 expression increased in L-NAT pretreated irradiated cells compared to irradiated cells. Hence, L-NAT could be a potential radioprotective that may inhibit oxidative stress and DNA damage and maintain mitochondrial health and Ca^2+^ levels by activating p-ERK1/2 and p53 expression in Neuronal cells.

## 1. Introduction

The biological response to ionizing radiation (IR) in the central nervous system (CNS) is not understood convincingly. The adverse effects of radiation exposure are directly proportional to the quality and dose of ionizing radiation [1]. Nuclear and radiological catastrophes may have devastating consequences for people, the environment, and the economy. Chernobyl (USSR, 1986), Goiania (Brazil, 1987), and Fukushima Daiichi (2011, Japan) were all terrible disasters that demonstrated how devastating these calamities may be. The Centers for Disease Control and Prevention (CDC) report that Cardiovascular/Neurovascular syndrome frequently manifests after a higher dose exposure (20 Gy-50 Gy) [2, 3]. On the other hand, radiation is the mandatory modality for treating patients with primary brain tumors, intracranial malignancies, and brain metastases. However, radiation therapy significantly induced adverse effects on surrounding normal tissues, mainly by inducing inflammation and radio-necrosis in the CNS [4]. It has been observed that about 50-90% of patients may progressively suffer from cognitive dysfunction after radiation therapy [5]. Therefore, radiation doses of more than 16 Gy delivered as a single fraction, 24 Gy given in three fractions, and more than 27.5 Gy given in five fractions has been advised in clinical practice to enhance local cavity management, particularly in patients with radioresistant tumors [6]. Professional employees occupational exposed to radiation in radiodiagnostic and radiotherapy centers are at risk (due to increased DNA damage, oxidative stress, and defect in blood count), as shown in previous works [7, 8]. To manage radiation toxicity, radiobiologists are endeavouring to produce non-toxic radioprotectors capable of preventing acute radiation syndrome (ARS) caused by radiation exposure. No therapeutic medicine is available anywhere in the world for treating ARS, especially Neurovascular syndrome, at doses more than 10 Gy [3]. Ionizing radiation's effects were orchestrated through the direct destruction and oxidation of crucial biomolecules (DNA, proteins, and lipids). The neuronal tissues contain high levels of polyunsaturated fatty acids, which make them highly susceptible to oxidative damage. Radiation-induced oxidative stress may persist longer in the central nervous system [9]. Radiation can generate reactive oxygen species (ROS) by direct radiolysis of cellular water through NADP^+^, NADPH, and the mitochondrial electron transport system [10]. Consequently, gamma radiation enhanced the activation of cytochrome oxidase, lipid peroxidation, mitochondrial membrane potential, and ATP production [11, 12]. Radiation causes decreased antioxidant enzymes such as superoxide dismutase (SOD), catalase, glutathione (GSH), glutathione S-transferase subunit (GSTs), and glutathione peroxidase (GPx) activities. It thus disturbs redox homeostasis in the cellular milieu [13]. Mitochondria are vital regulators for cell survival or death after ionizing radiation exposure. Irradiation may alter mitochondrial membrane potential in the nerve cells, which may aberrantly disrupt mitochondrial functioning, including cellular metabolism, redox homeostasis, Ca^2+^ balancing, and apoptosis [14, 15]. Ionizing radiation activates mitochondrial biogenesis for higher ATP production that may be utilized by nerve cells for DNA damage response (DDR) and apoptosis induction *via* cytochrome-c and caspase-3 pathways [12, 16].

Radiation-induced inflammation in the neurological system is regulated by releasing several neuropeptides, including substance P (11 amino acid peptide). Substance P is an agonist of the neurokinin-1 receptor that, upon activation, increases inflammation and apoptosis in irradiated cells and tissues [17, 18]. The neurokinin-1 receptor (NK-1R) antagonist, N-acetyl-L-tryptophan (L-NAT), has been reported to suppress cytochrome-c release and caspase-3 activation in neuronal cells [19]. N-acetyl-L-tryptophan has a well-established role as a neuroprotectant in treating Parkinson's disease, ALS, and other neurodegenerative diseases [19, 20]. Our group has recently reported that N-acetyl-L-tryptophan may neutralize Ochratoxin-A mediated oxidative stress in human embryonic kidney cells [21]. Nevertheless, in the present study, N-acetyl-L-tryptophan is being reported for the first time as a radioprotector against ionizing radiation in neuronal cells. The present study investigated the efficacy of N-acetyl-L-tryptophan towards ionizing radiation using Neuro2a cells. Radiation-induced cytotoxicity and its counter-balancing by N-acetyl-L-tryptophan pre-treatment to irradiated cells were studied. The radioprotective efficiency of N-acetyl-L-tryptophan was assessed in terms of cell survival, redox regulation, mitochondrial Ca^2+^ homeostasis and membrane potential modulation capabilities, direct or indirect DNA damage prevention, and apoptosis inhibition abilities. The experimental evidence reported in the present study will provide new insight into radiation toxicity in the neuronal system. N-acetyl-L-tryptophan can be a radioprotector, especially for the neurological system. It can be utilized for the benefit of cranial radiotherapy patients to protect the surrounded normal tissues and prevent acute radiation syndrome (ARS).

## 2. Materials and Methods

### 2.1. Reagents

Propidium iodide (PI), High glucose Dulbecco Modified Eagle Medium (HG-DMEM), and RNase and Fetal bovine serum (FBS) were obtained from Sigma-Aldrich, St Louis, MO, USA. Ethanol was purchased from Merck India Pvt. Ltd, Mumbai, India. Disodium hydrogen phosphate (Na_2_HPO_4_.2H_2_O) and Dimethyl sulfoxide (DMSO) was purchased from Central Drug House, New Delhi, India. 3-(4,5-dimethyl-2-yl)-2,5-diphenyltetrazolium bromide (MTT) and Phosphate-buffered saline (PBS) were procured from Himedia Laboratory Pvt. Ltd, Mumbai, India. 3,8-Diamino-5ethyl-6-phenylphenanthridinium bromide (EtBr) 2',7'-Dichlorodihydroflurescein diacetate (DCF-DA) and 6-amino-9-(2-methoxycarbonylphenyl xanthan-3-ylidene azanium chloride (Rhodamine-123) was purchase from Calbiochem, Merck India Pvt. Ltd, Mumbai, India. MitoSOX, Mitotracker Green, Mitotracker Red, ER-tracker Blue-white DPx, and Rhod-2 AM were procured from Thermo Fisher Scientific, USA. Primary antibodies were procured, such as Caspase-3 (Calbiochem), ERK-1/2, p-ERK-1/2 (Thermo Fisher Scientific, USA), gamma-H2aX (Sigma), p53 (Santa Cruz Biotechnology), were procured whereas, AP Conjugate secondary antibodies produced against mouse procured from Abgenex and secondary antibodies produced against rabbit procured from Sigma-Aldrich, St Louis, MO, USA. Calcium assay kit (Elabsciences, USA), Measure-iT High sensitivity Assay Kit (M36051; Molecular Probes, Invitrogen, USA), and mouse MPO Elisa Assay Kit (Fine test, China).

### 2.2. Cell Culture

Neuro2a cells were purchased from National Centre for Cell Science NCCS (Pune, India). Neuro2a cells were routinely cultured in DMEM containing high glucose, sodium bicarbonate (2.20 g/L), HEPES buffer (2.38 g/L), and sodium pyruvate (110 mg/L) and 10% heat-inactivated FBS with 5000 U penicillin at 37°C under 5% CO_2_ and 95% humidity. Cells were passaged every 2-3 days to maintain them in a healthy log phase. For experiments, cells were cultured at low density (0.1 X 10^5^cells/ml) in 60 mm tissue culture dishes (BD Pharmingen, Franklin Lakes, NJ), and all experiments were performed after cells had achieved 70% confluence.

### 2.3. Irradiation

Neuro2a cells were treated with L-NAT (dissolved in serum-free DMEM-HG) for 3 h before *γ*-irradiation using Co^60^ gamma-source (Bhabhatron-II, Panacea, Medical Technologies Pvt. Ltd. Bangalore, India) with a dose rate ranging from 0.8 to 0.676 Gy/h at room temperature.

### 2.4. Cell Survival Analysis in the Context of Cellular Metabolic Viability

Cell survival of Neuro2a cells in terms of metabolic viability was determined by performing MTT assay [22]. The following four experimental groups were formed to analyze the radioprotective effect of L-NAT in Neuro2a cells:

Gp1: Untreated control group of Neuro2a cells.

Gp2: Irradiated (4-70 Gy) group of Neuro2a cells.

Gp3: L-NAT treated group of cells; Neuro2a cells were treated with different concentrations (1.95-1000 *μ*g/ml) of L-NAT.

Gp4: L-NAT (0.001-10 *μ*g/ml) pretreated (-1 h to -4 h) plus irradiated (20 Gy) group of Neuro2a cells.

In brief, 4000 cells/well were seeded in the 96-well plates for 24 h. The metabolic activity of Neuro2a cells divided into four experimental groups was assessed, as described below, by incubating the cells with 5 mg/ml MTT for 2.5 h. After that, removed free MTT solution and formed purple formazan crystals were solubilized by adding 100 *μ*l of DMSO solution for 15 min. The absorbance of the purple-colored complex solution was recorded at 570/670 nm wavelength.

### 2.5. Estimation of the Radioprotective Effect of L-NAT Using SRB Assay

The radioprotective efficacy of L-NAT was determined in Neuro2a cells using SRB assay [23]. Neuro2a cells were seeded at 4000 cells/well in 96 well plates for 24 h. Then cells were pretreated (-3 h) with different concentrations of L-NAT (0.001-10 *μ*g/ml) with irradiation (20 Gy). After 72 h of incubation, cells were fixed with 10% TCA for 1 h, then subjected to washing with distilled water 3 times. After that, cellular proteins were stained with sulforhodamine (0.02% SRB in 1% acetic acid) for 1 h at room temperature. Further, 1% acetic acid was used to wash the cells 3 times and dry them overnight. 10 mM Tris (pH 10) dissolved the cell-SRB complex. The absorbance of the colored complex formed was recorded at 560 nm.

### 2.6. Evaluation of the Radioprotective Effect of L-NAT Using Colony-Forming Ability Assay

Exponentially growing cells were plated in triplicates in 6-well tissue culture dishes at a uniform density of 200 cells/well. After 24 h of incubation, Neuro2a cells were either irradiated with different doses of gamma radiation (2-7 Gy) or pretreated (-3 h) with L-NAT at different concentrations (0.02-0.08 *μ*g/ml) and then irradiated (5 Gy). These cells were incubated for 10 days in new media at 37°C in CO_2_ (5%). After incubation, 70% methanol was used to fix the colonies. Further, 1% crystal violet (dissolved in 70% methanol) was used for staining and colony counting [24].

### 2.7. Microscopic Analysis of Neuro2a Cells

Morphological analysis of Neuro2a cells upon L-NAT and gamma radiation treatment was performed using fluorescence microscopy. Cells were pretreated (-3 h) with L-NAT (0.01-0.08 *μ*g/ml) and then treated with gamma radiation (20 Gy) and incubated for 72 h at 37°C. After completion of the incubation period, cells were visualized under an inverted fully automatic fluorescent microscope (10X) (Nikon Ti, Japan) using phase contrast mode to assess the morphological features, neurite outgrowth, and presence of apoptotic, necrotic bodies or cellular debris. Mitochondrial ROS and mass (MitoSOX Red and Mitotracker Green) were also assessed amongst experimental groups at (40X) (Nikon Ti, Japan) using FITC/TRIC mode.

### 2.8. Determination of DNA Damage Using the Comet Assay

A modified neutral assay developed by Ostling and Johanson was used to evaluate DNA damage using the comet assay [25]. Briefly, as previously mentioned, DNA damage was measured in the control and all treatment groups. After 24 h of incubation, the cells were harvested in PBS. The cell suspension (5 *μ*l) (total number of ~10,000 cells) was mixed with 95 *μ*l of pre-warmed 0.4% ultra-low agarose and formed a layer on microscopic slides pre-coated with 1% agarose. These slides were submerged in lysis buffer (25 mM ethylene-diamine tetra-acetic acid, 1% sodium sarcosinate, and 2.5% sodium dodecyl sulfate, pH 10.5) for 20 min at 4°C and electrophoresed (buffer: 2.5 mM ethylene-diamine tetra-acetic acid, 90 mM boric acid, and 90 mM Trizma base, pH 8.3) at 370Am or 24 V for 30 min at 4°C. After a brief rinse in neutralizing buffer, the comet was stained with ethidium bromide (in phosphate buffered saline pH 7.4) for 5 min and analyzed under a bright field microscope (10X) (Nikon Ti, Japan) using TRIC mode.

### 2.9. Estimation of Radioprotective Effect of L-NAT against *γ*-Radiation-Induced Cell Death in Neuro2a Cells

Cell death in Neuro2a cells was assessed by PI (propidium iodide) uptake versus exclusion. Neuro2a cells were plated in 60 mm culture dishes for 24 h. Cells were pretreated (-3 h) with L-NAT (0.01-0.08 *μ*g/ml) and then gamma irradiation (20 Gy). After 72 h, Neuro2a cells were harvested, and PBS was washed by centrifugation at 250 X g for 5 min at 4°C. Pellets were suspended in PBS (0.5 ml) premixed with propidium iodide (PI; 5 *μ*g/ml). Samples were stored on ice, and flow cytometric analysis was performed using a BD FACS Aria III USA equipped for acquisition at a rate of 10,000 cells per sample using the BD FACS Diva Software for analysis [26]. In order to create the bar diagram, the mean fluorescence intensity (MFI) of a cell population compared to the control was determined using the fluorescence intensity of the cells caused by PI uptake (Ex/Em was reported at 493/636 nm).

### 2.10. Determination of Intracellular Redox Levels

2,7-dichlorofluorescein diacetate (H_2_-DCFDA) probe was used to determine radiation-induced intracellular free radical redox potential [27]. Briefly, as mentioned above, radiation-induced oxidative stress was assessed in control and all treatment groups as previously mention above. The generated intracellular free radicals were estimated by incubating the cells with H_2_-DCFDA (5 *μ*M) at 37°C for 25 min at different time intervals (0.5-48 h). Cells were harvested, and PBS washed by centrifugation at 250 X g for 5 min at 4°C. As previously described above, samples were acquired and analyzed in the control and all treatment groups. The fluorescence signal of the cells due to trapped DCF (excitation/emission = 485/535 nm) was analyzed as the mean fluorescence intensity (MFI) of a cell population with respect to control, which was used to plot the bar diagram.

### 2.11. Determination of Mitochondrial Redox, Mitochondrial Mass, Mitochondrial Ca^2+^ Levels, and Endoplasmic Reticulum Stress in Neuro2a Cells upon Irradiation and L-NAT Pretreatment

In brief, the irradiated mitochondrial redox status was estimated in L-NAT pretreated Neuro2a cells using the MitoSOX Red mitochondrial superoxide indicator (M36008, Molecular Probe). The following four experimental groups were created and kept the same for the rest of the experiments:

Gp1: Untreated control Neuro2a cells.

Gp2: Neuro2a cells irradiated gamma radiation (20 Gy).

Gp3: Neuro2a cells treated with L-NAT (0.04 *μ*g/ml).

Gp4: Irradiated (20 Gy) Neuro2a cells pretreated (-3 h) with L-NAT (0.04 *μ*g/ml).

After all treatments, mitochondrial ROS generation was estimated by incubating cells with MitoSOX Red (5 *μ*M) at 37°C for 25 min. The fluorescence signal of MitoSOX Red has 510/580 nm, excitation/emission. However, the mitochondrial mass in irradiated (20 Gy) and L-NAT pretreated Neuro2a cells was estimated by incubation with Mitotraker green (1 *μ*M) at 37°C for 25 min. The fluorescence signal of MitoTracker Green has 490/516 nm, excitation/emission, FITC channel. The mitochondrial Ca^2+^ of irradiated and L-NAT pretreated Neuro2a cells was assessed by incubation with Rhod 2 AM (500 nM) at 37°C for 25 min. The fluorescence signal of Rhod-2 AM has 552/581 nm, Ex/Em, PE channel. Similarly, endoplasmic reticulum stress was measured using a specific ER tracker Black/White DPx probe (5 *μ*M) by incubating at 37°C for 25 min. ER tracker Black/White DPx probe has Ex/Em 374/430-640 nm and DAPI as filters. After incubation with these dyes (MitoSOX Red, MitoTracker Green, Rhod 2 AM, and ER tracker Black/White DPx probe), cells were harvested and washed twice with HBSS buffer by centrifugation at 3500 rpm for 5 min at 4°C. As described above, samples were acquired and analyzed in the control and all treatment groups. The fluorescence signal of the probe was analyzed as the mean fluorescence intensity (MFI) of a cell population with respect to the control, which was used to plot the bar diagram. In contrast, the analysis of the fluorescence signal was kept the same for all the experiments.

### 2.12. Measurement of Mitochondrial Membrane Potential (MMP)

Rhodamine-123, a specific fluorescent probe, measured the mitochondrial membrane potential. As previously stated, radiation-induced depolarization of mitochondrial membrane potential was measured in the control and all treatment groups. Cells were harvested and washed twice with PBS by centrifugation at 250 X g for 5 min at 4°C. Rhodamine-123 was added to cells with a working concentration of 1 *μ*g/ml in PBS for 15 min incubation at 37°C. After that, cells were washed and incubated in EtBr (40 ng/ml; 0.5 ml PBS) for 30 min at 37°C for dual fluorescent staining and stored on ice. As previously described above, the acquisition and analysis of samples were performed in the control and all treatment groups. Fluorescence was detected at 485/535 nm wavelength [28, 29].

### 2.13. Antioxidant Assay

#### 2.13.1. Estimation of Catalase Assay (CAT)

Catalase enzyme activity was determined for all four-treatment groups using the Aebi method. A reaction mixture of catalase assay was prepared with 50 mM phosphate buffer (750 *μ*l), 15 mM H_2_O_2_ (250 *μ*l), and 20 *μ*l cell Lysate. The absorbance of the reaction mixture was recorded at 240 nm for 1 min at 15 seconds intervals in both blank and test samples [30, 31].

#### 2.13.2. Estimation of Superoxide Dismutase (SOD) Activity

The superoxide dismutase enzyme activity was measured in all four experimental groups using the Marklund and Marklund method. The reaction mixture containing 30 *μ*l cell Lysate, tris-HCl buffer (pH 8.2), 2 mM pyrogallol, 30 mM EDTA (250 *μ*l). The absorbance of the reaction mixture was recorded at 420 nm for 3 min at 30 seconds intervals in both blank and test samples [30, 31].

#### 2.13.3. Estimation of Reduced Glutathione (GSH) Activity

Reduced glutathione (GSH) activity was determined using the Beutler method. Sample was prepared according to previous described protocol. The reaction mixture was prepared with Na_2_HPO_4_ buffer (600 *μ*l), Ellmens reagent (250 *μ*l), and 250 *μ*l cell Lysate. Absorbance was recorded at 412 nm [30, 31].

### 2.14. Evaluation of Nitrite Concentration on L-NAT Pretreated and Irradiated Neuro2a Cells

The effect of L-NAT (0.04 *μ*g/ml) pretreatment on nitrite concentration in irradiated (20 Gy) cells was estimated using the Measure-iT High-sensitivity nitrite assay kit (M36051, Molecular Probe). According to the manufacturer protocol provided with the kit, the nitrite concentration of Neuro2a cells was evaluated at different time intervals (2, 4, 24, and 48 h). The fluorescence was measured at 365/450 nm by a spectrofluorimeter (Synergy BIO-TEK Instrument USA).

### 2.15. Evaluation of Myeloperoxidase (MPO) Activity in Irradiated and L-NAT pretreatedNeuro2a Cells

The effect of L-NAT pretreatment on myeloperoxidase activity in irradiated Neuro2a cells was evaluated using the myeloperoxidase (MPO) Elisa Kit (EM0010, Fine Test). As previously stated, Neuro2a cells were divided into four experimental groups. According to the manufacturer protocol provided with the kit, and MPO activities were assessed at different times in each group (i.e., 2, 4, 24, and 48 h). The absorbance was measured at 450 nm by a spectrofluorometer (Synergy BIO-TEK Instrument USA).

### 2.16. Estimation of the Amount of Calcium in the Cytosol of Neuro2a Cells

Intracellular calcium levels in Neuro2a cells were assessed using a calcium assay kit (Elabscience; E-BC-K103-M). The amount of calcium in the cytosol of Neuro2a cells were assessed at different times in each group (i.e., 2, 4, 24, and 48 h), as per the manufacturer protocol. The absorbance was measured at 610 nm by spectrofluorometer (Synergy BIO-TEK Instrument USA).

### 2.17. Immunoblotting

As described in the previous section, cells from all treatment groups were harvested just after 48 h of irradiation and PBS washed by centrifugation at 5000 rpm for 5 min at 4°C. The cell pellet was re-suspended in RIPA buffer and sonicated at 1.5 min for 3 times. The supernatant was collected by centrifugation at 14000 rpm for 25 min at 4°C. Protein estimation was performed using the Bradford method, and proteins were resolved on a 10% SDS-PAGE gel. Then, proteins were transferred to a nitrocellulose membrane blot using wet transfer technology. Nitrocellulose membrane blot was blocked with filter 3% BSA as a blocking buffer. Afterward, the blots were transferred to a solution of primary monoclonal antibodies (1 : 1000 dilution) overnight at 4°C. Further, blots were washed with TBST buffer and transferred to a secondary antibody (1 : 10,000 dilution) conjugated with AP (Alkaline-Phosphatase) for 2 h. The blots were washed 3 times with TBST buffer, and BCIP/NBT solution, premix, was used to develop protein expression signatures on the blots. Quantitative analysis of blots was performed using ImageJ software. We use total protein as housekeeping proteins (HKPs) and ponceau staining to detect total protein in the sample [32, 33]. Total proteins were used for normalization in western blots because of alteration in the expression of actin, tubulin, and GAPDH housekeeping proteins in response to ionizing radiation, similar to the previous finding in different stress conditions [33–35].

### 2.18. Statistical Analysis

All data were expressed as mean ± SEM of triplicated experiments. The statistically significant variation between different groups was determined by one-way ANOVA (analysis of variance) followed by a Tukey's *post hoc* test, and a Student's unpaired t-test was used in the western blot analysis. *P* value (*p* <0.05) was considered significant.

## 3. Result

### 3.1. Radioprotective Efficacy of L-NAT Assessed in Neuro2a Cells

#### 3.1.1. Neurotoxic Effect of L-NAT on the Neuro2a Cells

**Figure 1:**
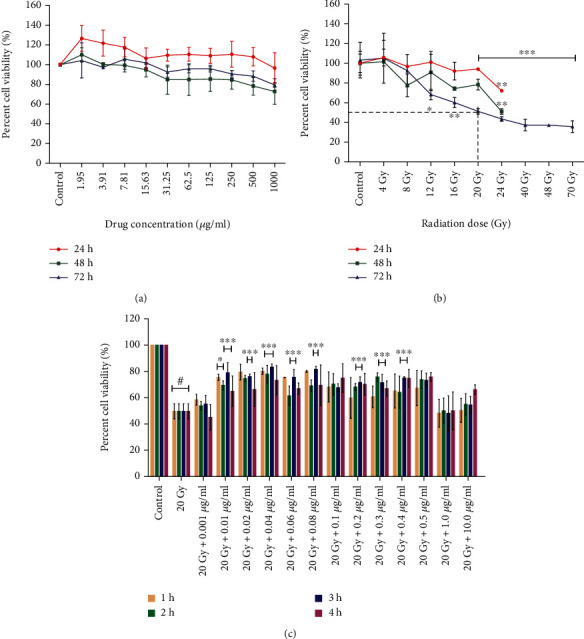
Determination of radiosensitivity of Neuro2a cells and the radioprotective activities of L-NAT using MTT assays. (a) The toxicity effect of L-NAT in Neuro2a cells was measured at different concentrations of L-NAT (1.95-1000 *μ*g/ml) at different times (24-72 h). (b) Radiation-induced sensitization in Neuro2a cells was assessed using different radiation doses (4-70 Gy) at variable time intervals. (c) The radioprotective efficacy of L-NAT against gamma radiation (LD_50_; 20 Gy) was estimated in Neuro2a cells. Statistical significance was calculated using one-way ANOVA with Turkey, and the *p*-value was presented as ^∗^*p* <0.05; ^∗∗^*p* <0.01; ^∗∗∗^*p* <0.001 with respect to irradiated control. ^**#**^p <0.001 with respect to control.

Different concentrations (1.95-1000 *μ*g/ml) of L-NAT were used to estimate its probable toxicity (LD_50_/LD_90_) in Neuro2a cells. After 24 hours, the maximum metabolic activity (126.64%) was observed at the lowest L-NAT concentration (1.95 *μ*g/ml) as compared to untreated control cells (Figure 1(a)). A modest decrease in metabolic activity in Neuro2a cells was observed after 48-72 h at concentrations of >15.63 *μ*g/ml L-NAT. However, even at the highest L-NAT concentration (1000 *μ*g/ml), the LD_50_ concentration was not achieved at any time interval (24-72 h) (Figure 1(a)). These findings suggest that L-NAT promotes metabolic activity in Neuro2a cells during the first 24 h and does not induce significant neurotoxicity at higher concentrations.

#### 3.1.2. Sensitization of Neuro2a Cells to Gamma Radiation

Observations of present study were demonstrated an ascending reduction in the survival of Neuro2a cells with increasing radiation doses (4-70 Gy). However, 20 Gy radiation dose was calculated as the LD_50_ dose (51.27% survival; p <0.001%) for Neuro2a cells at 72 h as compared to the untreated control group (Figure 1(b)). These findings suggest that Neuro2a cells show radioresistance behavior to high-dose ionizing radiation.

#### 3.1.3. L-NAT Pretreatment Confers Radioprotective Potential against Gamma Radiation

Significant protection against gamma radiation in Neuro2a cells was observed at mostly all tested concentrations of L-NAT, however, maximum radioprotection (p <0.001%), i.e., 83.19% survival, was achieved with irradiated Neuro2a cells that were pretreated (-3 h) with 0.04 *μ*g/ml concentration of L-NAT as compared to irradiated (20 Gy; LD_50_) cells that not pretreated with L-NAT (Figure 1(c)).

#### 3.1.4. Radiation-Induced Growth Inhibition and Its Amelioration by L-NAT Pretreatment to Neuro2a Cells

**Figure 2:**
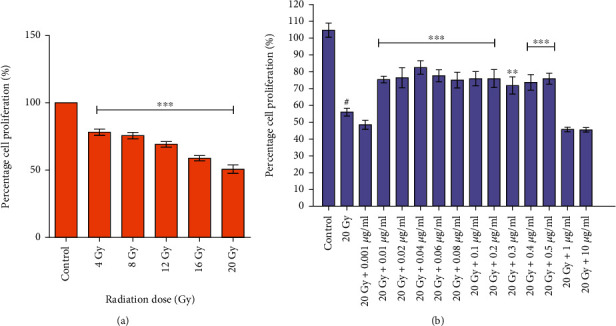
Determination of radioprotective activities of L-NAT in terms of reproductive survival against ionizing radiation using SRB assays. (a) Effect of radiation on cellular reproducibility in Neuro2a cells was analyzed at different radiation doses (4-20 Gy) at 72 h. (b) L-NAT pretreatment was assessed for cellular reproducibility in Neuro2a cells against gamma radiation (LD_50_; 20 Gy) with different concentrations of L-NAT (0.001-10 *μ*g/ml) at 72 h. Error bars are mean ± SEM of triplicate measurement (n =3). Statistical significance was calculated using one-way ANOVA with Turkey and the *p*-value presented as ^∗^*p* <0.05; ^∗∗^*p* <0.01; ^∗∗∗^*p* <0.001 with respect to irradiated control. ^**#**^p <0.001with respect to control.

Results of the SRB experiment showed that gamma radiation treatment (4-20 Gy) significantly (p <0.001%) inhibited the growth of Neuro2a cells compared to unirradiated cells. Similar to the MTT assay (Figure 1(b)), the SRB assay identified 20 Gy radiation dose as the LD_50_ (50.69% growth inhibition; p <0.001%) for Neuro2a cells (Figure 2(a)). Additionally, it was shown that L-NAT pretreatment (-3 h) of irradiation (20 Gy) cells maintained significantly (p <0.001%) high proliferation, namely 82.52% at 0.04 *μ*g/ml concentration, in comparison to irradiated cells without pretreatment with L-NAT (Figure 2(b)). These findings overwhelmingly support the excellent radioprotective properties of L-NAT against gamma radiation-induced cell death.

#### 3.1.5. L-NAT Pretreatment Confers Inhibition in Reproductive Cell Death against Ionizing Radiation in Neuro2a Cells

**Figure 3:**
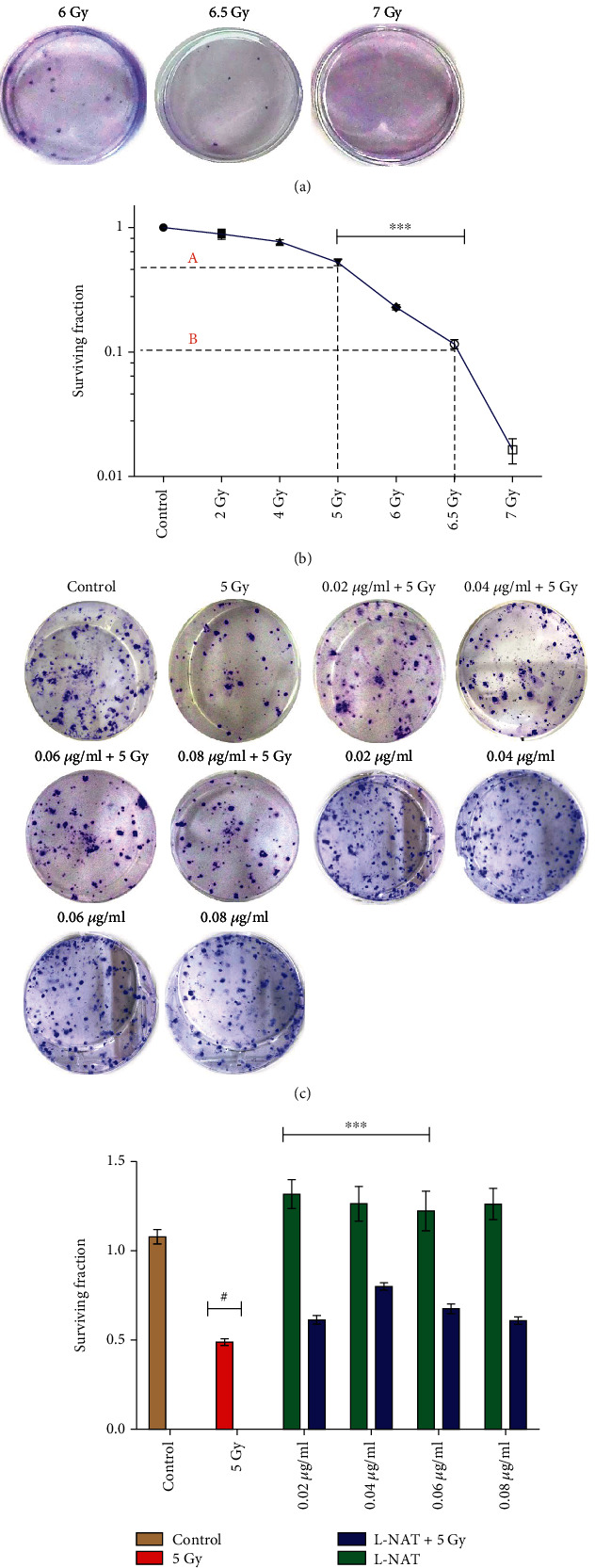
Estimation of radioprotective properties of L-NAT against ionizing radiation in Neuro2a cells using colony-forming units (CFU) assay. (a, b) Ionizing radiation dose response curve of Neuro2a cells at different radiation doses (2-7 Gy) was established. (c, d) The radioprotective effectiveness of L-NAT in irradiated Neuro2a cells was calculated at indicated radiation dose (5 Gy: LD_50_). Statistical significance was calculated using one-way ANOVA with Turkey and the *p*-value presented as ^∗^*p* <0.05; ^∗∗^*p* <0.01; ^∗∗∗^*p* <0.001 with respect to irradiated control. ^#^p <0.001with respect to control.

The colony-forming ability of Neuro2a cells was observed to be >80% when cultured without any treatment under normal growth conditions. Effect of radiation on colony forming ability of Neuro2a cells at different radiation doses (2-7 Gy) was determine and LD_50_ was calculated to be 5 Gy, while radiation dose LD_90_ was estimated to be 6.5 Gy (Figures 3(a) and 3(b)). Only L-NAT treatment at different concentrations (0.02-0.08 *μ*g/ml) exhibited steady colony-forming units corresponding to the survival of the cells (132%-126%; Figures 3(c) and 3(d)). Neuro2a cells were pretreated (-3 h) with L-NAT and then irradiated with gamma radiation (5 Gy) to determine L-NAT's radioprotective efficacy via a colony-forming unit after one week of incubation was counted. A maximum of ~80% (p <0.001) cell survival in terms of the appearance of the colony-forming unit was observed with irradiated Neuro2a cells that were pretreated (-3 h) with L-NAT (0.04 *μ*g/ml) on the 10^th^ day as compared to the cells only irradiated but not pretreated with L-NAT (Figure 3(d)). These findings demonstrated the reproductive death inhibition activity of L-NAT in irradiated cells.

#### 3.1.6. Radiation-Induced Cell Death and Its Inhibition by L-NAT Pretreatment to Neuro2a Cells

**Figure 4:**
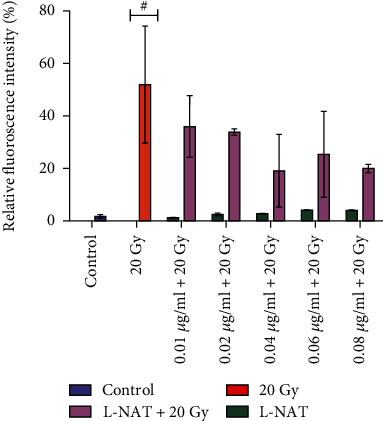
The effect of L-NAT pretreatment on gamma radiation-induced cell death in Neuro2a cells. The radioprotective effect of L-NAT (0.01-0.08 *μ*g/ml) on radiation-induced cell death was determined using propidium iodide (PI) uptake assay by Flow cytometry. Values were expressed as mean ± SEM, where *p*-value presented as ^∗^*p* <0.05; ^∗∗^*p* <0.01; ^∗∗∗^*p* <0.001 with respect to irradiated control. ^#^p <0.001 with respect to control.

The results of the present study demonstrated that gamma irradiation (20 Gy) induced the death of 51.98% (p <0.01%) of cells as compared to unirradiated control cells. On the other hand, L-NAT (0.04 *μ*g/ml) pretreatment (-3 h) to the irradiated cells significantly ameliorated apoptotic/necrotic cell death (~19.17%) at 72 h as compared to irradiated cells without pretreatment with L-NAT (Figure 4). These findings strongly demonstrated the excellent radioprotective properties of L-NAT in Neuro2a cells against lethal dose of gamma radiation.

### 3.2. Mechanism of Radioprotection Offered by L-NAT

#### 3.2.1. Radiation-Induced DNA Damage and Its Prevention by L-NAT Pretreatment

**Figure 5:**
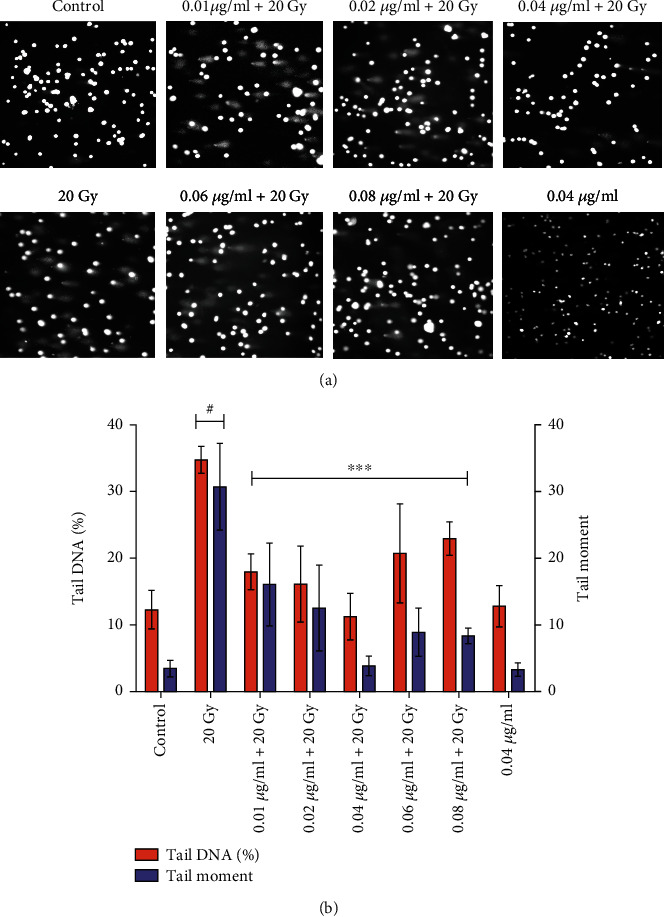
Protective influence of L-NAT pretreatment on radiation-induced DNA damage in Neuro2a cells. (a) Microscopic examination of radiation-induced DNA damage and its prevention by L-NAT pretreatment. (b) The effect of L-NAT (0.01-0.08 *μ*g/ml) pretreatment on radiation-induced DNA damage in Neuro2a cells was investigated using the comet assay. Values were expressed as mean ± SEM, where *p*-value presented as ^∗^*p* <0.05; ^∗∗^*p* <0.01; ^∗∗∗^*p* <0.001 with respect to irradiated control. ^**#**^p <0.001 with respect to control.

It was also observed that L-NAT (0.04 *μ*g/ml) pretreatment (-3 h) to irradiated cells significantly contributes to the reduction of DNA contents in the comet tail and tail length at 24 h as compared to irradiated cells without pretreatment with L-NAT (Figures 5(a) and 5(b)). L-NAT (0.04 *μ*g/ml) pretreatment (-3 h) to irradiated cells was found to inhibit the comet tail moment by 3.87% and DNA content in comet tail by 11.26% as compared to irradiated (20 Gy) cells that have 34.75% DNA in the comet tail and 30.71% tail movement (p <0.001, Figure 5(b)) at 24 h. The microscopic analysis also showed that the irradiation (20 Gy) group of cells has a larger number of comets than the L-NAT (0.04 *μ*g/ml) pretreatment (-3 h) irradiated cells (Figure 5(a)). As a consequence, the current study found that pretreatment with L-NAT of irradiated Neuro2a cells effectively protects DNA from radiation-induced damage and hence significantly contributes to the protection of Neuro2a cells from radiation-induced cell death.

#### 3.2.2. Microscopic Observations on Radiation-Induced Neurite Outgrowth of Neuro2a Cells and Its Modulation by L-NAT Pretreatment

**Figure 6:**
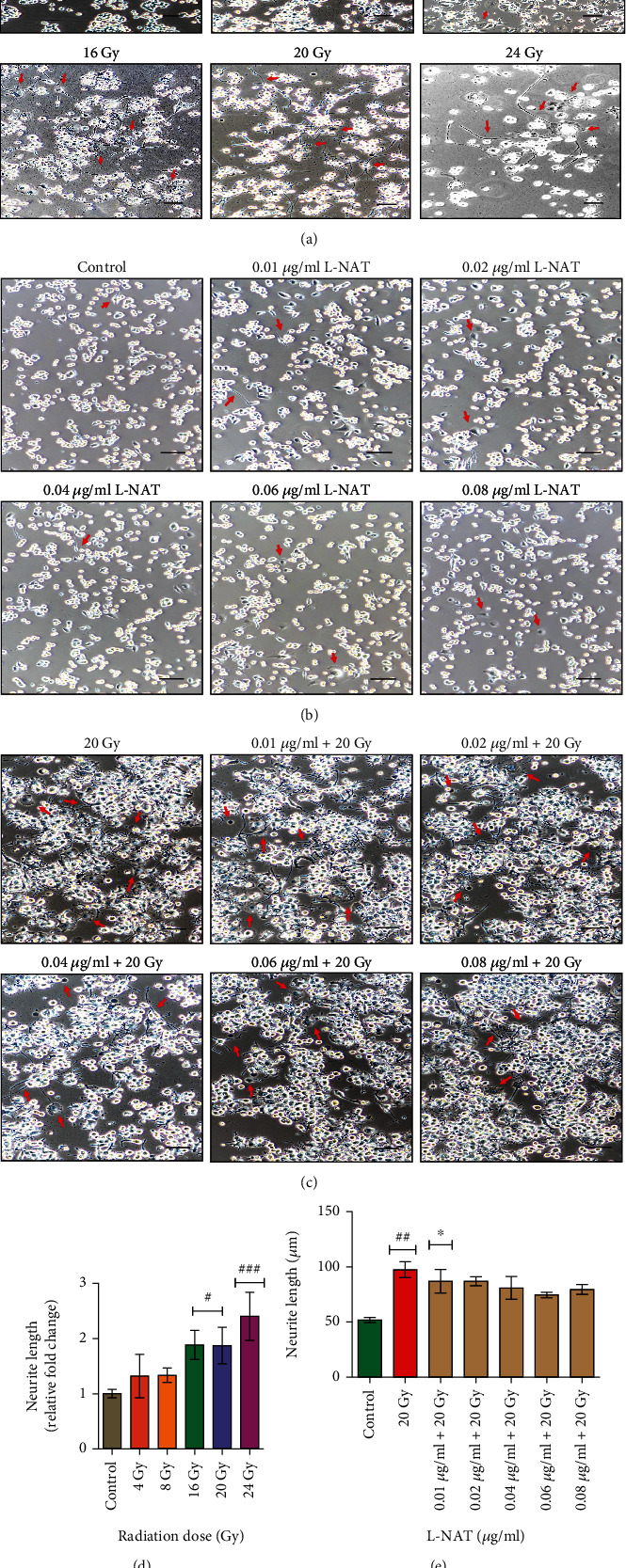
Gamma radiation mediated neurite outgrowth induction in Neuro2a cells and its inhibition by L-NAT pretreatment to irradiated cells. (a) Phase contrast micrographs (10x magnification; Nikon Ti, Japan) were demonstrated radiation dose (4-24 Gy) dependent neurite outgrowth in Neuro2a cells. (b) The effect of L-NAT pretreatment (0.01-0.08 *μ*g/ml) on neurite outgrowth development in non-irradiated and (c) irradiated Neuro2a cells was analyzed post 72 h. Each scale bar represents 100 *μ*m. (d) Quantitative analysis of radiation doses (4-24 Gy) dependent neurite outgrowth of Neuro2a cells. Arrow marks represent neurite outgrowth and swollen/shrunken cells. (e) Quantitative analysis of L-NAT pretreatment on radiation-induced neurite outgrowth of Neuro2a cells. Error bars are mean ± SEM of triplicate measurement (n =3). Statistical significance was determined and *p*-value presented as ^**#**^*p* <0.05; ^**##**^*p* <0.01; ^**###**^*p* <0.001 with respect to control. ^∗^*p* <0.05; ^∗^*p* <0.01; ^∗∗∗^*p* <0.001 with respect to irradiated control.

Microscopic investigation revealed a dose-dependent increase in neurite outgrowth in Neuro2a cells exposed to gamma radiation (4-24 Gy) compared to controls (Figure 6(a)). After 72 h, a maximal increase (2.41 fold) in neurite outgrowth was observed at a 24 Gy dose of gamma radiation compared to control (p <0.001; Figure 6(d)). Furthermore, the overall size of Neuro2a cells was observed to be enhanced as compared to controls, most likely due to cytoplasmic swelling and inflammation. L-NAT treatment (0.01-0.08 *μ*g/ml) had no direct harmful impact on cell swelling or neurite outgrowth in Neuro2a cells compared to control (Figure 6(b)). L-NAT pretreatment (0.04-0.08 *μ*g/ml) of irradiation (20 Gy) Neuro2a cells resulted in a considerable reduction of neurite outgrowth compared to irradiated cells without L-NAT pretreatment (Figures 6(c) and 6(e)). However, the maximum reduction (~74.56 *μ*m ±2.54 *μ*m and 79.50 *μ*m ±4.59 *μ*m) in neurite outgrowth was observed at 0.06 *μ*g/ml and 0.08 *μ*g/ml, respectively, pretreated L-NAT concentration as compared to irradiated cells (20 Gy; 97.41 *μ*m) at 72 h (p <0.001; Figure 6(e)).

#### 3.2.3. Effect of L-NAT Pretreatment to Neutralized Gamma Radiation-Induced Redox Imbalance in Neuro2a Cells

**Figure 7:**
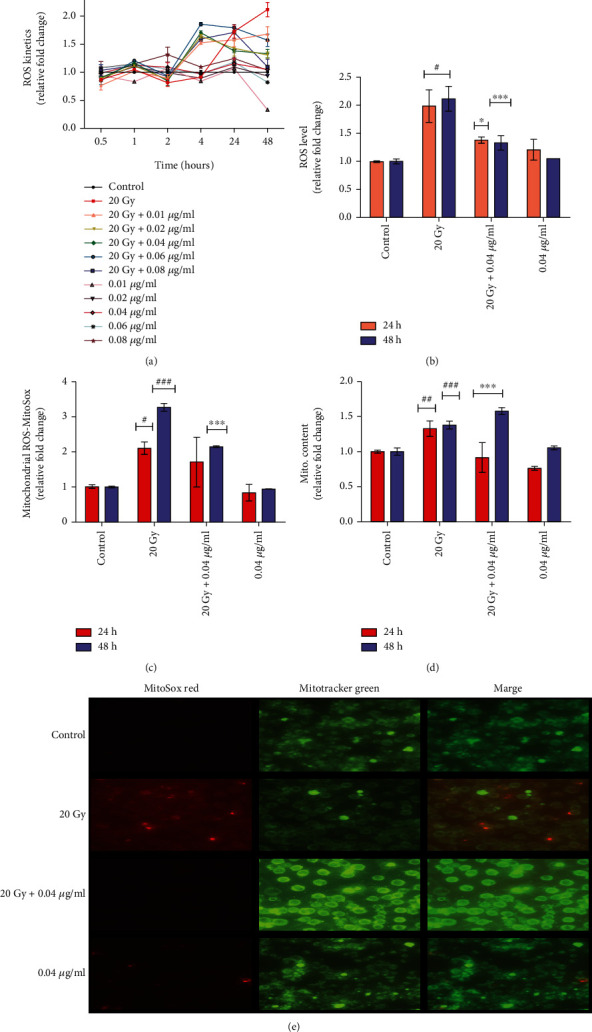
Evaluation of L-NAT pretreatment reduces oxidative stress in terms of Reactive Oxygen Species (ROS) generation in irradiated Neuro2a cells. (a) ROS kinetics under the influence of gamma irradiation and L-NAT (0.01-0.08 *μ*g/ml) pretreatment to Neuro2a cells were evaluated by measuring H_2_-DCFDA (5 *μ*M) level in cells at different time points (0.5-48 h). (b) L-NAT pretreatment (0.04 *μ*g/ml) was found to contribute to reducing ROS levels in irradiated (20 Gy) Neuro2a cells at 24-48 h. (c) L-NAT pretreatment significantly reduced Mitochondrial ROS in irradiated Neuro2a cells with regulating mitochondrial content (d) as observed at 24-48 h. (e) The neutralizing effect of L-NAT on radiation-induced mitochondrial ROS formation and mitochondrial content in Neuro2a cells were observed under a Fluorescence microscope (Nikon Ti, Japan; 40x magnification) at 48 h. Error bars are mean ± SEM of triplicate measurement (n =3). Statistical significance was determined and *p*-value presented as ^∗^*p* <0.05; ^∗∗^*p* <0.01; ^∗∗∗^*p* <0.001 with respect to irradiated control, ^#^p <0.001 with respect to control.

No significant increase in intracellular ROS was found in irradiated (20 Gy) Neuro2a cells at early time intervals, i.e., up to 4 h, as compared to controls (Figure 7(a)). Further observations revealed that radiation exposure significantly (p <0.001%) increased ROS levels up to 1.99 fold at 24 h and 2.12 fold at 48 h as compared to control Neuro2a cells (p <0.001; Figures 7(a) and 7(b)). Interestingly, it was observed that L-NAT (0.01-0.04 *μ*g/ml) pretreatment (-3 h) to irradiated (20 Gy) Neuro2a cells significantly contributed to inhibiting endogenous ROS. Though, maximum decrease (~1.38 fold and 1.33 fold; p <0.001) in ROS was evident at 24-48 h, respectively, at 0.04 *μ*g/ml concentration of L-NAT in irradiated cells as compared to irradiated cells without pretreatment with L-NAT (Figures 7(a) and 7(b)).

A significant increase (~2.11-3.27 fold; p <0.01%) in mitochondrial ROS was observed at 24-48 h with irradiated (20 Gy) Neuro2a cells as compared to control (Figure 7(c)). However, L-NAT (0.04 *μ*g/ml) pretreatment (-3 h) to irradiated cells led to a significant decrease (~1.71-2.15 fold; p <0.001%) in mitochondrial ROS levels at 24-48 h as compared to irradiated Neuro2a cells that were not pretreated with L-NAT (Figure 7(c)). Further, a molecular probe, Mitotracker Green, was used to determine the effect of gamma radiation on mitochondrial mass. A significant increase (~1.3 fold; p <0.001%) in mitochondrial mass was observed in irradiated (20 Gy) Neuro2a cells at 24-48 h as compared to the control cells (Figure 7(d)). However, L-NAT (0.04 *μ*g/ml) pretreatment (-3 h) to irradiated Neuro2a cells led to a decrease in mitochondrial mass up to ~0.92-1.58 fold (p <0.001%) at 24-48 h as compared to the irradiated control that was not pretreated with L-NAT (Figure 7(d)). Further, microscopic analysis again confirmed the previous findings by demonstrating that L-NAT (0.04 *μ*g/ml) pretreatment (-3 h) to irradiated cells may ameliorate mitochondrial ROS and mitochondrial mass *via* probing the ROS using MitoSox Red and mitochondrial mass using MitoTracker Green probe, respectively (Figure 7(e)). L-NAT (0.04 *μ*g/ml) pretreatment (-3 h) followed by irradiation (20 Gy) caused a decrease in the fluorescence intensity of MitoSox Red along with Mito Tracker Green, suggested a reduction in mitochondrial ROS and mitochondrial mass, respectively, as compared to irradiated cells without pretreatment with L-NAT (Figure 7(e)).

#### 3.2.4. Mitochondrial Membrane Potential Disturbance in Irradiated Neuro2a Cells and Its Regulation by L-NAT Pretreatment

**Figure 8:**
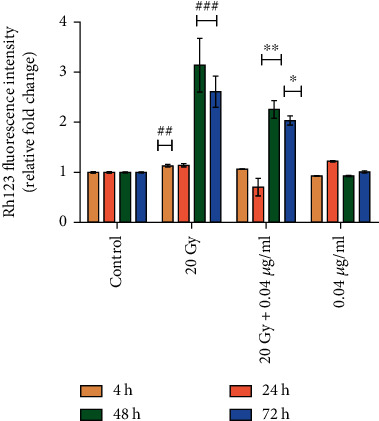
Effect of L-NAT pretreatment on mitochondrial membrane potential stabilization in irradiated Neuro2a cells. Ionizing radiation-induced depolarization of the mitochondrial membrane potential and L-NAT pretreatment help to stabilize mitochondrial membrane potential in irradiated Neuro2a cells. Error bars are mean ± SEM of triplicate measurement (n =3). Statistical significance was determined and *p*-value presented as ^∗^*p* <0.05; ^∗∗^*p* <0.01; ^∗∗∗^*p* <0.001 with respect to irradiated control, ^**#**^p <0.001with respect to control.

Ionizing radiation leads to a significant (p <0.001) increase in mitochondrial membrane potential (MMP) up to ~3.14 fold at 48 h and 2.61 fold at 72 h as compared to control (Figure 8). Nevertheless, ionizing radiation was not found to disrupt mitochondrial membrane potential (MMP) at early time points (i.e., 4 and 24 h), suggesting that Neuro2a cells resist radiation-induced oxidative stress at early time points. However, it was observed that irradiated cells that were pretreated (-3 h) with L-NAT (0.04 *μ*g/ml) led to maximum (2.26-3.14 fold; p <0.001) restoration of membrane potential at 24-48 h as compared to irradiated cells without pretreatment with L-NAT (Figure 8).

#### 3.2.5. Influence of L-NAT Pretreatment on Gamma Radiation-Induced Perturbations Cytosolic and Mitochondrial Ca^2+^ Homeostasis and Associated Endoplasmic Reticulum (ER) Stress in Neuro2a Cells

**Figure 9:**
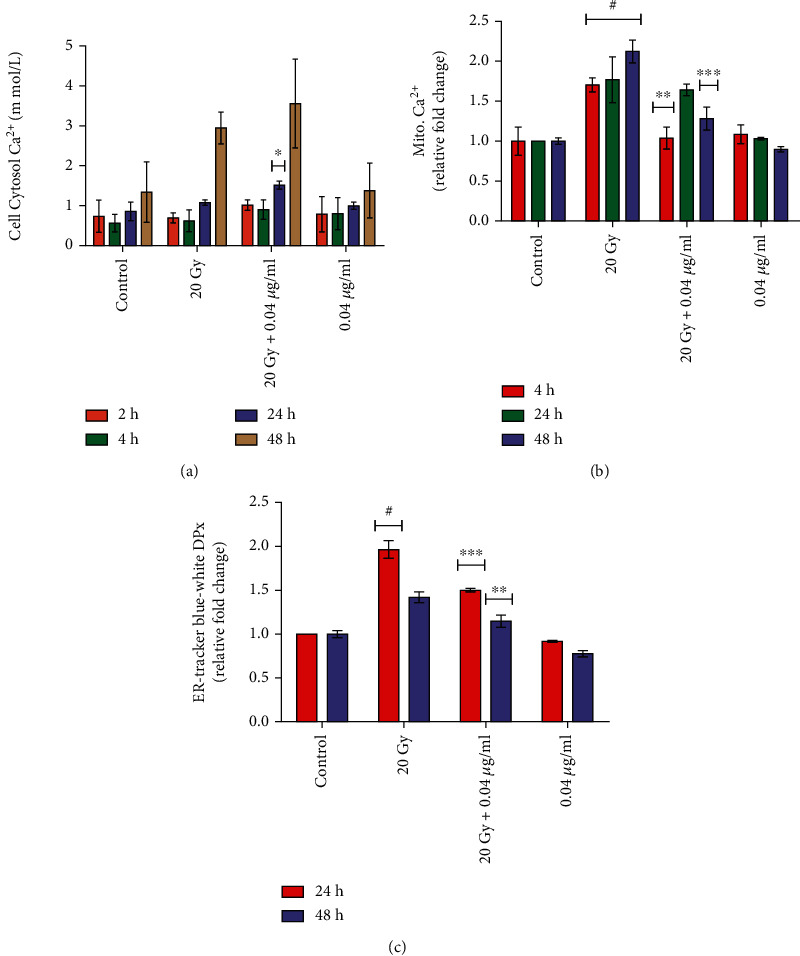
Role of L-NAT pretreatment on calcium homeostasis regulation in irradiated Neuro2a cells. (a) L-NAT pretreatment (0.04 *μ*g/ml) regulates cytosolic calcium levels in irradiated Neuro2a cells at time points (4-48 h). (b) L-NAT pretreatment to irradiated cells indicated reduced mitochondrial calcium levels compared to irradiated cells. (c) Apart from calcium homeostasis, L-NAT pretreatment also regulates endoplasmic reticulum stress as observed using ER-specific ER tracker Black/White DPx probe (5 *μ*M) in Neuro2a cells. Error bars are mean ± SEM of triplicate measurement (n =3). Statistical significance was determined and *p*-value presented as ^∗^*p* <0.05; ^∗∗^*p* <0.01; ^∗∗∗^*p* <0.001 with respect to irradiated control,^**#**^p <0.001with respect to control.

Significantly increased in cytosolic calcium content up to 1.08-2.95 mmol/L at 24-48 h was observed in irradiated (20 Gy) Neuro2a cells as compared to control (Figure 9(a)). Interestingly, L-NAT pretreatment (0.04 *μ*g/ml) to irradiated cells led to an increase in cytosolic calcium content up to 1.51-3.56 mmol/L (p <0.05) at 24-48 h as compared to irradiated cells (Figure 9(a)). To further verify the observations, calcium concentrations in mitochondria were estimated using a specific mitochondrial calcium detection probe, Rhod 2 AM (1 *μ*M). The results of the study demonstrated a significant (p <0.001%) increase (1.71, 1.77, and 2.13 fold at 4, 24, and 48 h, respectively) in Ca^2+^ concentration in irradiated (20 Gy) Neuro2a cells as compared to untreated control cells (Figure 9(b)). Most interestingly, a significant (p <0.001%) decrease in mitochondrial Ca^2+^ concentration (i.e., 1.04, 1.64, and 1.28 fold at 4, 24, and 48 h, respectively) was noticed with irradiated (20 Gy) Neuro2acells that were pretreated (-3 h) with L-NAT (0.04 *μ*g/ml) as compared to irradiated cells without pretreatment with L-NAT (Figure 9(b)). The detailed experimental observations of the present study proved that L-NAT pretreatment to irradiated Neuro2a cells is involved in the accumulation of cytosolic calcium, although at the same time playing a role in reducing the accumulation of calcium in the mitochondrial matrix. One of the possible reasons, L-NAT acts as a blocker of mitochondrial calcium channels/pumps, e.g., voltage-dependent anion channels (VDACs) or mitochondrial calcium uniporter (MCU). The endoplasmic reticulum (ER) is the primary source of Ca^2+^ released in the cytosol under various stresses, including radiation stress. Therefore, the possible role of L-NAT pretreatment in IR-induced endoplasmic reticulum (ER) stress was analyzed using the ER tracker Black/White DPx probe (5 *μ*M). The results of this study demonstrated a significant (p <0.001%) increase (1.96-1.42 fold at 24-48 h) in endoplasmic reticulum (ER) stress compared to control (Figure 9(c)). On the other hand, L-NAT (0.04 *μ*g/ml) pretreatment (-3 h) to irradiated Neuro2a cells was found to be instrumental in decreasing endoplasmic reticulum (ER) stress up to 1.5 fold (p <0.001%) at 24 h and 1.15 fold (p <0.001%) at 48 h (Figure 9(c)) as compared to only irradiated cells. In conclusion, Ca^2+^ homeostasis studies suggested that L-NAT pretreatment may prevent Ca^2+^ migration from the endoplasmic reticulum into the cytosol and inhibit Ca^2+^ transportation from the cytosol to the mitochondrial matrix in irradiated cells.

#### 3.2.6. Radiation Reduces Superoxide Dismutase (SOD) Activities, and L-NAT Treatment Maintains Activities

**Figure 10:**
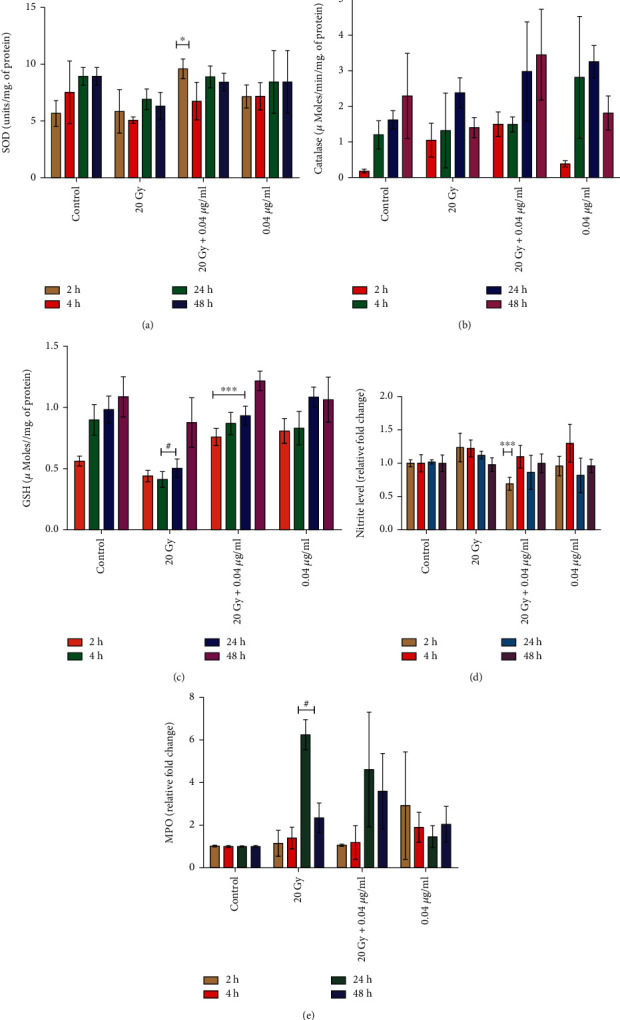
L-NAT mediated cellular enzymatic and non-enzymatic antioxidant machinery modulation in irradiated Neuro2a cells. (a) L-NAT pretreatment (0.04 *μ*g/ml) to irradiated Neuro2a cells increased SOD and (b) catalase expressions at time points (2-48 h). (c) L-NAT pretreatment significantly increased GSH level, thus reducing oxidative stress in irradiated Neuro2a cells at time points (2-24 h). (d) L-NAT pretreatment regulated the nitrite level mildly and reduced RNS stress in irradiated Neuro2a cells at time points (2-48 h). (e) Considerably decrease in MPO level was evident with L-NAT pretreatment plus irradiated cells as compared to irradiated cells at 4 h and 24 h, respectively. Error bars are mean ± SEM of triplicate measurement (n =3). Statistical significance was determined and p-value presented as ^∗^p <0.05; ^∗∗^p <0.01; ^∗∗∗^p <0.001 with respect to irradiated control; #p <0.001 with respect to control.

Results of the study were indicated a significant decrease in SOD activity upon irradiation (20 Gy) at 4-48 h in Neuro2a cells compared to the control. Whereas, L-NAT pretreatment (0.04 *μ*g/ml) to irradiated (20 Gy) cells was found to maintain SOD levels in Neuro2a cells as compared to only irradiated cells (Figure 10(a)).

#### 3.2.7. Influence of L-NAT Pretreatment on Catalase Activity of Irradiated Neuro2a Cells

A significant decrease in catalase activity in Neuro2a cells was noticed at 48 h upon irradiation (20 Gy) compared to the control. However, L-NAT pretreatment (0.04 *μ*g/ml) to irradiated (20 Gy) Neuro2a cells was found to increase (2.98 *μ*Moles/min/mg. and 3.45 *μ*Moles/min/mg. of protein at 24 h and 48 h, respectively) catalase activity at 4-48 h as compared to irradiated cells without pretreatment with L-NAT (Figure 10(b)). These findings suggested that L-NAT pretreatment to irradiated cells may counter-balance intracellular ROS levels in irradiated Neuro2a cells and thus protect them from radiation-induced oxidative stress.

#### 3.2.8. Radiation Reduced Endogenous Reduced Glutathione (GSH) Levels and Its Prevention by L-NAT Pretreated in Neuro2a Cells

Significant (p <0.001) decrease in reduced glutathione (GSH) levels in irradiated (20 Gy) Neuro2a cells was observed at all-time points (2-48 h) as compared to control (Figure 10(c)). However, L-NAT pretreatment (0.04 *μ*g/ml) to irradiated (20 Gy) cells was found to contribute significantly (p <0.001) to an increase in reduced glutathione (GSH) levels at different time intervals (2-48 h) as compared to irradiated cells without pretreatment with L-NAT (Figure 10(c)). Therefore, by increasing GSH levels, L-NAT pretreatment may decrease oxidative damage in the irradiated Neuro2a cells.

#### 3.2.9. L-NAT Pretreatment Confers Nitrite Level Modulation in Irradiated Neuro2a Cells

A mild increase in nitrite levels in irradiated Neuro2a cells at 2-48 h was observed in irradiated cells compared to control. However, as the function of L-NAT pretreatment (0.04 *μ*g/ml), nitrite levels were found to be subsidized significantly (p <0.001%; at 2 h) in irradiated cells as compared to irradiated cells without pretreatment with L-NAT (Figure 10(d)).

#### 3.2.10. Radiation Causes Increase Myeloperoxidase Activities and Its Inhibition by L-NAT Pretreatment in Neuro2a Cells

A significant (6.25 fold; p <0.05%) increase in myeloperoxidation in irradiated Neuro2a cells was observed at 24 h as compared to control (Figure 10(e)). L-NAT pretreatment to irradiated Neuro2a cells significantly decreased (~4.62 fold) myeloperoxidase activities at 24 h compared to irradiated cells without pretreatment with L-NAT (Figure 10(e)). It was suggested that L-NAT pretreatment to irradiated Neuro2a cells might contribute to significantly inhibiting myeloperoxidation/lipid peroxidation.

#### 3.2.11. Inhibition of Intrinsic Apoptosis through Modulation of Caspase-3, *γ*-H2aX, p53, ERK-1/2, and P-ERK1/2 Expression upon L-NAT Pretreatment to Irradiated Neuro2a Cells

**Figure 11:**
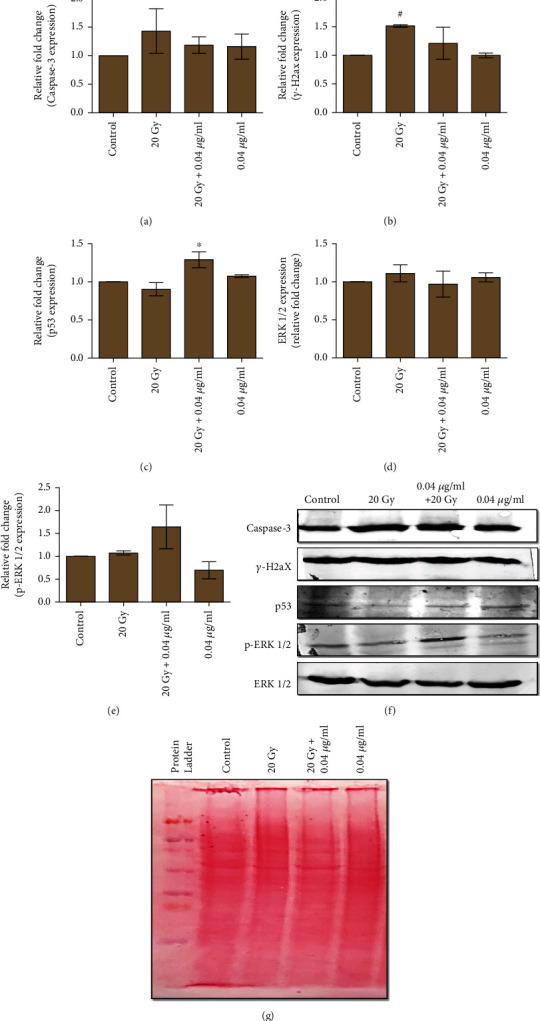
Analysis of apoptosis regulating protein expression in L-NAT pretreated and irradiated Neuro2a cells. Quantitative analysis of protein expression of Caspase-3 (a), *γ*-H2aX (b), p53 (c), ERK-1/2 (d) and p-ERK-1/2 (e) was performed in Neuro2a cells after L-NAT and gamma radiation treatment. (f) Western Blot representative images of Caspase-3, *γ*-H2ax, p53, ERK-1/2, and p-ERK-1/2 were provided. (g) Ponceau staining was also performed to detect the total protein present in the samples. Statistical significance was calculated using Student's unpaired t-test and *p*-value presented as ^∗^*p* <0.05; ^∗∗^*p* <0.01; ^∗∗∗^*p* <0.001 with respect to irradiated control, ^#^p <0.001with respect to control. NOTE: Ponceau staining of total proteins was used as a positive control in this study because standard housekeeping proteins (HKPs), i.e., GAPDH, tubulin, and actin, were found modulated by irradiation in the Neuro2a cells (Data not shown).

Results of the present study was demonstrated moderate to significant inhibition in the caspase-3 (1.18 fold) and *γ*-H2aX protein (1.21 fold) in L-NAT (0.04 *μ*g/ml) pretreated (-3 h) plus irradiated (20 Gy) Neuro2a cells as compared to only irradiated cells that were not pretreated with L-NAT (Figures 11(a), 11(b), 11(f)) and 11(g)). However, a significant increase in p53 (~1.29 fold; p <0.05%) and p-ERK1/2 expression (~1.65 fold) was observed with irradiated Neuro2a cells that were pretreated with L-NAT as compared to irradiated cells without pretreatment with L-NAT (Figures 11(c), 11(e), 11(f) and 11(g)). The present findings suggested that L-NAT pretreatment contributes immensely to protecting irradiated Neuro2a cells by enhancing DNA repair and thus cell survival.

## 4. Discussion

Ionizing radiation induces significant deleterious effects on the central nervous system (CNS) [36, 37]. Therefore, one of the primary concerns is the development of radioprotectors and radiomitigators to alleviate radiation-mediated cytotoxicity in the CNS. For the first time, N-acetyl-L-tryptophan (L-NAT) was evaluated for its radioprotective efficacy in Neuro2a cells. Irradiated Neuro2a cells pretreated with N-acetyl-L-tryptophan exhibited substantial radioprotection in terms of metabolic viability (83.19%; Figure 1(c)), reproductive viability (82.52%; Figure 2(b)), colony-forming survivability (80%; Figure 3(d)), and ameliorating apoptotic/necrotic cell death (19.17%; Figure 4) against lethal dose of gamma radiation. These findings suggested the excellent radioprotective activity of N-acetyl-L-tryptophan in the cells of neuronal origin. N-acetyl-L-tryptophan glucoside (NATG), a close glucosidic derivative of NAT, has been reported for its radioprotective properties in the murine macrophage j774A.1 cells and mice against lethal radiation dose, supported the present study [38, 39]. Ionizing radiation augments oxidative stress *via* the induction of free radicals in the cellular milieu that causes direct or indirect DNA damage, leading to genomic instability [40]. The comet assay results demonstrated significant DNA damage supported by high DNA content in the comet tail in irradiated cells but L-NAT pretreatment significantly reduced comet tail length and respective tail DNA content (Figures 5(a) and 5(b); p <0.001). Therefore, it seems that L-NAT pretreatment to irradiated Neuro2a cells reduces radiation-induced DNA damage. Microscopic observations suggested that L-NAT pretreatment to irradiated (20 Gy) Neuro2a cells inhibited neurite outgrowth and may prevent radiation-induced differentiation of Neuro2a cells as well as protect young, immature neurons from radiation-induced cell death (Figures 6(a)–6(e)). Ionizing radiation has been reported to enhance neurite outgrowth, the cell cycle G2 arrest, and decrease the viability of the cells in a dose-dependent manner (4, 8 or, 16 Gy) in Neuro2a cells, provided a gain support to the present study [41].

Mitochondrial function modulation is an important strategy for achieving radioprotection in the neuronal and other biological systems such as GI, hematopoietic, and hepatic [12, 42–44]. The results of L-NAT treatment with irradiation in Neuro2a cells showed a significant reduction in ROS levels in the cellular milieu and mitochondrial ROS compared to irradiated cells without pretreatment with L-NAT (Figures 7(a)–7(c)). These observations suggested that L-NAT treatment may efficiently reduce ROS and thus inhibit oxidative stress in irradiated cells. Ionizing radiation-induced oxidative stress causes hypo/hyperpolarization of the mitochondrial membrane that promotes the leakage of radicals and thus activates mitochondrial oxidative stress and intrinsic apoptosis pathways [45]. L-NAT pretreatment, however, was observed to reduce the hyperpolarization of the mitochondrial membrane in Neuro2a cells exposed to radiation (Figure 8), possibly by reducing reactive oxygen species in the cellular milieu or preventing the accumulation of cytoplasmic Ca^2+^ in the mitochondrial matrix. L-NAT treatment with irradiated Neuro2a cells showed lower accumulation of Ca^2+^ in the mitochondrial matrix as compared to the cytoplasm, suggested that, L-NAT may act as a blocker of mitochondrial calcium channels/pumps, e.g., voltage-dependent anion channels (VDACs) or mitochondrial calcium uniporter (MCU) that inhibits migration of cytosolic Ca^2+^ into the mitochondrial matrix (Figures 9(a) and 9(b)). However, Gamma irradiation stimulates ER stress, as observed using the Blue-White DPX probe in Neuro2a cells that are found subsidized upon L-NAT pretreatment (Figure 9(c)). These observations suggested that L-NAT significantly maintains ER stress and Ca^2+^ homeostasis between the cytosol and the mitochondrial matrix and protects cells from radiation-induced apoptosis. Interestingly, L-NAT treatment to un-irradiated Neuro2a cells was found to enhance mild ROS levels compared to control at 24 h, which was subsidized at a subsequent time, i.e., 48 h (Figure 7(b)). These observations suggest a priming phenomenon initiating in Neuro2a cells after L-NAT administration, increasing hydrogen peroxide and other ROS/RNS levels in the cellular milieu. The priming phenomena/intrinsic mild oxidative stress may further activate the endogenous antioxidant machinery to combat oxidative stress in the advance that may likely arise in the near future due to irradiation, as shown in the previous study [21, 38, 39]. Similarly, L-NAT treatment increased mild hyperpolarization of mitochondrial membrane, antioxidant enzyme activity such as catalase, SOD, MPO, and non-enzymatic factors, such as GSH and nitrite levels (RNS) compared to control in Neuro2a cells, were confirm the effect of intrinsic oxidative stress on the activation of endogenous antioxidant machinery (Figures 8 and 10). The observations of the present investigation demonstrated a significant increase in catalase, SOD, and GSH, in irradiated Neuro2a cells that were pretreated with L-NAT compared to irradiated cells that were not pretreated with L-NAT (Figures 10(a)–10(c)). However, it was observed that a significant increase in MPO activities and nitrite levels in irradiated Neuro2a cells and L-NAT pretreated reduced radiation-induced MPO activities and nitrite levels (Figures 10(d)–10(e)). It has been found that L-NAT negatively regulated H_2_O_2_ conversion into hypochlorous acid (HOCl) and contributed to cells' protection against radiation-induced DNA damage and cell death by lowering MPO levels in irradiated cells.

Protein expression analysis demonstrated L-NAT pretreatment to irradiated cells significantly decreased the expression of caspase-3 and *γ*-H2aX proteins compared to irradiated cells without pretreatment with L-NAT (Figures 11(a), 11(b), 11(f) and 11(g)). The present findings suggested that L-NAT pretreatment to irradiated cells may contribute to the amelioration of apoptosis by inhibiting the apoptotic pathways. In the current study, ionizing radiation leads to a decrease in p53 expression compared to control that was enhanced upon L-NAT pretreatment to the irradiated Neuro2a cells (Figures 11(c), 11(f) and 11(g)). Similarly, previous studies found a significant decrease in p53 expression at 8 Gy radiation dose, supporting the present investigation [41]. Pretreatment with L-NAT to irradiated Neuro2a cells led to enhanced p53 expression as compared to irradiated cells (1.29 fold; p <0.05; Figures 11(c), 11(f) and 11(g)), further justified a prominent role of p53 in DNA repair and cell cycle arrest, which provides crucial time for the activation of various DNA-repair systems [46, 47]. Pretreatment with L-NAT to irradiated Neuro2a cells showed a significant reduction in DNA damage compared to irradiated cells in the comet assay and a decrease in *γ*-H2aX protein expression that may correlate with an increase in p53 activity (Figure 5(b) and 11(b)–11(c)). A significant increase in p-ERK1/2 protein expression without any change in non-phosphorylated ERK1/2 protein expression was observed in irradiated cells pretreated with L-NAT compared to irradiated cells without pretreatment with L-NAT (~1.65 fold; Figures 11(d)–11(g)). These observations suggested that as a function of L-NAT pretreatment to irradiated Neuro2a cells, increased phosphorylation of ERK1/2 protein may further activate the ERK1/2 signaling pathway and thus contribute to radioprotection. The activation of ERK 1/2 pathways that promote cell survival supports the present investigation [48]. However, L-NAT is a well-known NK-1 receptor antagonist, and future studies will explore the potential role of NK-1 receptors in L-NAT-mediated radioprotection.

## 5. Conclusion

**Figure 12:**
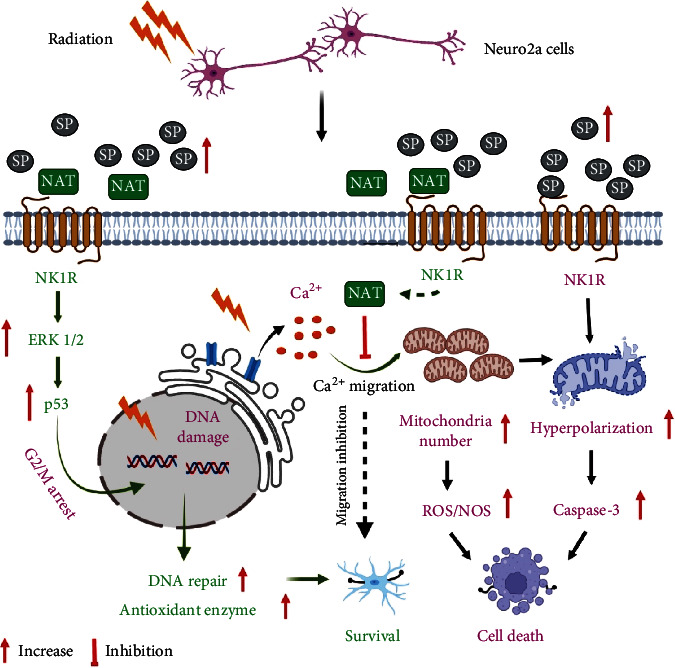
A summarized diagrammatic illustration to demonstrate the possible role of L-NAT in radioprotection. L-NAT binds with NK-1R and inhibits Substance P binding with its preferred receptor, i.e., NK-1R. The interaction of L-NAT with the NK-1R cellular system may be activated to maintain oxidative stress by reducing ROS, enhancing antioxidant enzymes activity, mitochondrial redox homeostasis, inhibit accumulation of Ca^2+^ ion concentration in the mitochondrial matrix, inhibiting apoptosis *via* inhibition of caspase-3 and *γ*-H2aX expression, as well as an increase in p53 and p-EKR1/2 expression that may contribute to achieving the desired level of radioprotection in Neuro2a cells.^# **#**^we use http://BioRender.com for creating these Diagrams.

The present study suggested that L-NAT pretreatment provides significant radioprotection while maintaining oxidative stress, mitochondrial redox homeostasis, the polarization of mitochondrial membrane potential, and inhibiting the migration of Ca^2+^ ions toward the mitochondrial matrix. There is a possibility that L-NAT acts as a blocker for mitochondrial calcium channels or pumps. L-NAT also increases antioxidant enzyme activity, enhancing DNA repair while inhibiting apoptosis through activating EKR1/2 and p53 signaling pathways (Figure 12).

## Data Availability

The authors confirm that the data supporting the findings of this study are available within the article.
